# *ZNF469* frequently mutated in the brittle cornea syndrome (BCS) is a single exon gene possibly regulating the expression of several extracellular matrix components

**DOI:** 10.1016/j.ymgme.2013.04.014

**Published:** 2013-07

**Authors:** Marianne Rohrbach, Helen L. Spencer, Louise F. Porter, Emma M.M. Burkitt-Wright, Céline Bürer, Andreas Janecke, Madhura Bakshi, David Sillence, Hailah Al-Hussain, Matthias Baumgartner, Beat Steinmann, Graeme C.M. Black, Forbes D.C. Manson, Cecilia Giunta

**Affiliations:** a Division of Metabolism, Connective Tissue Unit and Children's Research Center, University Children's Hospital, Zurich, Switzerland; b Genetic Medicine Research Centre, Institute of Human Development, Faculty of Medical and Human Sciences, The University of Manchester, UK; c St. Mary's Hospital, Central Manchester University Hospitals NHS Foundation Trust, Manchester Academic Health Science Centre, Manchester, UK; d Division of Human Genetics, Innsbruck Medical University, Innsbruck, Austria; e Department of Clinical Genetics, Children's Hospital at Westmead, Westmead NSW, Sydney, Australia; f King Khaled Eye Specialist Hospital, Division of Oculoplastics and Orbit, Saudi Arabia

**Keywords:** Brittle cornea, Keratoconus, Keratoglobus, *PRDM5* and *ZNF469*, Single exon gene, Genetic heterogeneity

## Abstract

Brittle cornea syndrome (BCS; MIM 229200) is an autosomal recessive generalized connective tissue disorder caused by mutations in *ZNF469* and *PRDM5*. It is characterized by extreme thinning and fragility of the cornea that may rupture in the absence of significant trauma leading to blindness. Keratoconus or keratoglobus, high myopia, blue sclerae, hyperelasticity of the skin without excessive fragility, and hypermobility of the small joints are additional features of BCS. Transcriptional regulation of extracellular matrix components, particularly of fibrillar collagens, by *PRDM5* and *ZNF469* suggests that they might be part of the same pathway, the disruption of which is likely to cause the features of BCS. In the present study, we have performed molecular analysis of a cohort of 23 BCS affected patients on both *ZNF469* and *PRDM5*, including those who were clinically reported previously [1]; the clinical description of three additional patients is reported in detail. We identified either homozygous or compound heterozygous mutations in *ZNF469* in 18 patients while, 4 were found to be homozygous for *PRDM5* mutations. In one single patient a mutation in neither *ZNF469* nor *PRDM5* was identified. Furthermore, we report the 12 novel *ZNF469* variants identified in our patient cohort, and show evidence that *ZNF469* is a single exon rather than a two exon gene.

## 1 Introduction

Brittle cornea syndrome (BCS; MIM 229200 and 614170229200614170) is a rare autosomal recessive connective tissue disorder with a severe ocular phenotype. Hallmarks of the condition include corneal thinning and fragility. The most striking feature of BCS is corneal rupture/perforation, either spontaneously or after minor trauma to the eye. Other ocular features include keratoconus, keratoglobus, high myopia and blue sclerae. Extraocular manifestations, which may be absent [2] include skin hyperelasticity in the absence of excessive fragility, joint hypermobility and kyphoscoliosis [1], [3], [4], [5], [6]. Hypercompliant tympanic membranes, developmental hip dysplasia, and large joint hypermobility were recently described as additional features of BCS [7]. The presence of clinical features overlapping with some forms of Ehlers Danlos Syndrome (EDS), particularly the kyphoscoliotic type of EDS (EDS VIA; MIM 225400), led to the suggestion that BCS and EDS VI were identical conditions [8]. EDS VIA is caused by the deficiency of the enzyme lysyl hydroxylase 1 (LH1) and the demonstration of normal LH1 activity in BCS confirmed the distinction between these two conditions [1], [6]. Since Stein et al. coined the term “brittle cornea syndrome” in 1968 [9], and a second description confirmed an autosomal recessive mode of inheritance [10], over 40 individuals have been clinically reported with the disease [1], [2], [7], [11], [12], [13], confirming that BCS is clinically distinct from EDS VIA and it presents a different natural history with a less severe prognosis.

Corneal rupture is the most frequent presenting feature of BCS, leading to blindness in nearly all cases. Prior to rupture, visual acuity in BCS patients is often affected by keratoconus and high myopia. The common combination of visual loss with hearing loss in BCS contributes to the serious functional impact of this disease on individuals and their families. It is likely that BCS remains under-diagnosed, particularly if concomitant connective tissue features are mild or absent. Yet an early diagnosis is desirable to make anticipatory management as effective as possible.

Brittle cornea syndrome is genetically heterogeneous and can be caused by mutations in either *ZNF469* (MIM 612078) [11] or *PRDM5* (MIM 614161) [7], [14]. Although *ZNF469* was reported as the first BCS causing gene in 2008, the mechanism by which mutations in it influence corneal development and structural integrity remains unknown [11]. Transcriptional regulation of extracellular matrix components, particularly fibrillar collagens, by *PRDM5* and *ZNF469* suggests that they might be part of the same pathway, the disruption of which is likely to cause the features of BCS [11], [15].

Recently, in order to test the hypothesis of a common pathomechanism of BCS, whether it arises from mutations in *PRDM5* or *ZNF469*, genome wide expression analysis was performed on RNA isolated from dermal fibroblasts from individuals with mutations in either *ZNF469* or *PRDM5* [7]. Several genes involved in the development and maintenance of the extracellular matrix (ECM) were found to be significantly down-regulated, and q-PCR of five of the most down-regulated genes (*COL4A1*, *COL11A1*, *EDIL3*, *HAPLN1* and *TGFβ2*) further supported this hypothesis [7].

Immunofluorescence staining of several extracellular matrix proteins was performed on *PRDM5* and *ZNF469* mutant fibroblasts. Indeed, disarray of collagens I and III, fibronectin and their receptor α2β1 and α5β1 integrins, further supported the hypothesis that PRDM5 and ZNF469 proteins regulate ECM organization through similar biochemical mechanisms [7]. In our present study the genes glypican 6 (*GPC6*), clusterin (*CLU*), thrombospondin I (*THBS1*) and procollagen C-endopeptidase enhancer 2 (*PCOLCE2*) which were found to be significantly altered in the two *ZNF469* mutants in our previous genome wide expression analysis, have been further investigated by qPCR in both *ZNF469* and *PRDM5* mutants, respectively. Glypican 6 (*GPC6*) is part of the heparan sulfate proteoglycan family, members of which acts as extracellular receptors for growth factors and interact with ECM proteins. Clusterin (*CLU*) is a multifunctional extracellular matrix chaperone and was of interest due to its involvement in stabilizing cell–matrix interactions and clearance of ECM degradation [16], [17], [18]. Thrombospondin I (*THBS1*) is a calcium-binding glycoprotein that interacts with a number of ECM molecules including collagen, fibronectin and integrin [19], [20], [21]. Procollagen C-endopeptidase enhancer 2 (*PCOLCE2*) is required for correct procollagen processing and deposition of collagen [22].

The *ZNF469* gene (NM_001127464) has previously been described as a 13 kb two exon gene located at 16q24.2 [11]. Recently, a role for *ZNF469* in normal corneal development has been suggested with several genome-wide association studies identifying this locus as a determinant of corneal thickness [23], [24], [25], [26]. In this article we report the results of the molecular investigations of 23 individuals from 13 unrelated families affected with BCS, including those who were clinically described previously [1]. The results clearly suggest that ZNF469 is the most frequent genetic cause of BCS: 12 novel sequence variants in *ZNF469*, and 3 *PRDM5* disease causing mutations that were previously presented in Burkitt Wrigth et al. [7] were identified. Furthermore we show evidence that *ZNF469* is a single exon rather than a two exon gene as previously annotated.

## 2 Methods

### 2.1 Biochemical analyses and dermal cell cultures

Total urinary pyridinolines were measured as described [27], [28] and were expressed as the ratio of lysyl pyridinoline (LP) to hydroxylysyl pyridinoline (HP).

Primary dermal fibroblast cultures were established from skin biopsies by routine procedures. Fibroblasts were routinely maintained in DMEM (Life Technologies, Paisley, UK), supplemented with 10% fetal calf serum at 37 °C, in 5% CO_2_. Fibroblasts were expanded until confluence, and then harvested by trypsin digestion.

### 2.2 Mutation analysis

Genomic DNA was isolated from peripheral blood leukocytes or cultured fibroblasts using standard techniques. Each single exon of *ZNF469* and subsequently of *PRDM5* was amplified by PCR using 5′ and 3′ primers designed on the flanking intron sequences as described [7], [11].

Mutation analysis by sequencing was performed using the Big-Dye Terminator cycle sequencing ready-reaction kit version 1.1 (Life Technologies) and an AB 3130xl Genetic Analyzer (Applied Biosystems, Life Technologies). Mutations in ZNF469 were referred according to the reference sequence NM_001127464 with the inclusion of the coding, 84bp-long, intronic sequence.

### 2.3 Expression study

Total RNA was extracted from cell lines using RNeasy Mini Kit and QIA Shredder (Qiagen, Crawley, UK). Microarrays were conducted on skin-derived fibroblasts from previously described patients 909_06, 915_07 [1] and two appropriate age and sex-matched controls using the Affymetrix Human Genome U133 Plus 2.0 array, according to the manufacturer's instructions (Affymetrix Inc, High Wycombe, UK) as previously described [7].

Molecular components of pathways highlighted in this analysis were further validated using quantitative real time polymerase chain reaction (qRT-PCR) on patients (BCS-001, BCS-002) [7], (909_6, 915_07) [1] and 4 control dermal fibroblast lines. Extracted total RNA was reverse-transcribed into single-stranded cDNA using a High Capacity RNA-to-cDNA Kit (Life technologies). The RT-PCR was performed using first-strand cDNA with TaqMan Fast Universal PCR Master Mix (Life Technologies). The assay numbers for the mRNA endogenous control (*GAPDH*) and target genes were as follows: *GAPDH* (Hs03929097_g1*), *Procollagen C-endopeptidase enhancer 2* (Hs00203477_m1), *Clusterin* (Hs00156548_m1), *Glypican 6* (Hs00170677_m1), and *Thrombospondin 1* (Hs00962908_m1). Quantitative PCR was performed on a StepOnePlus Real Time PCR system (Life Technologies). Quantitative PCR parameters for cycling were as follows: 50 °C incubation for 2 min, 95 °C for 10 min, 40 cycles of PCR at 95 °C for 15 s, and 60 °C for 1 min. All reactions were performed in a 10 μl reaction volume in triplicate. The mRNA expression level was determined using the 2^− Δ*C*^_t_ method.

One-way ANOVA and Bartlett's post-test using mean values and standard error were used to compare results from mutant and wild-type cells. These were performed on all the fold change means in all groups assessed (p < 0.0001), allowing the significance of multiple means to be assessed.

### 2.4 PCR to confirm *ZNF469* as a single exon gene

Total RNA extracted from two different control individuals was treated with DNase I (New England Biolabs Gmbh, Germany) to remove genomic DNA contaminants. RT-PCR was performed with Qiagen OneStep RT-PCR Kit (Qiagen, Hilden, Germany) according to the manufacturer's instructions, using primers spanning the whole putative intron: F — 5′C GGC ATG GAG TAC CAG TCG GAC A 3′ and R — 5′ C TTC CTG CCT CGC CGC CTT C 3′. Amplified fragments were cleaned using ExoSAP-IT® (Affymetrix, Inc.), sequenced on an AB 3130xl sequencer (Applied Biosystems, Life Technologies) as previously described, and analyzed with Sequence Pilot (JSI Medical Systems, GmbH, Germany).

## 3 Results

### 3.1 Clinical information

Clinical information was obtained through a questionnaire that was sent to each involved center and subsequently analyzed centrally. Clinical examination was performed by a geneticist, a pediatrician and an ophthalmologist. Diagnosis of BCS was based on clinical grounds in all index patients and proven by mutation analysis of *ZNF469* and *PRDM5*. Informed written consent from patients or their parents was obtained, in accordance with requirements of the Local Ethics Committees of the referring physicians.

Clinical data regarding BCS families 901–904, 908–910, 912, 914, and 915–918 was originally published by Al Hussain et al. [1] before the genetic basis of this condition was identified, while those of the newly diagnosed patients P1, P2, and P3 are described below and summarized in Table 1.

**Table 1:** The salient clinical findings of three novel affected individuals P1, P2 and P3 from unrelated families are indicated. ++ indicates highly significant feature; + indicates moderately significant feature; − indicates not present; N/A indicates data not available. **^#^**Note that P2 is the only compound heterozygous patient whereas all the others carry homozygous mutations.

	P 1	P2	P3
Age at diagnosis/gender	5 years/F	7 years/F	14 years/F
Origin	Indian	British	Pakistani
Consanguinity	Yes	No	Yes
*ZNF469*	**c.3476delG** p.Gly1159Alafs*105	**^#^c.[5788delC];[5788dupC]** p.[Gln1930Argfs*6];[Gln1930Profs*133]	**c.6444delG** p.Gln2149Serfs*51
Eyes			
Bluish sclera	++	++	++
Corneal rupture	++	++	++
Keratoconus/keratoglobus	++	N/A	++
Skin soft/hyperelastic	+	−	+
Joints			
Hypermobility of large joints	++	++	+
Hypermobility of small joints	++	++	++
Beighton score	8/9	N/A	4/9
Delayed motor development	+	−	+
Delayed cognitive development	−	−	+
Hearing impairment	++	++	−
Miscellaneous			
Fractures	−	−	+
Cardiovascular abnormalities	+	−	–

### 3.2 Patient 1 (P1)

Patient P1, aged 6 years and 3 months was the first child of first cousin parents originally from Andhra Pradesh in Eastern India. She had a 3 year old healthy brother and no family history of joint or ophthalmic problems. The family was multiply consanguineous.

The pregnancy was complicated by maternal hypertension but preceded to normal vaginal delivery at term. Birth weight was 3.2 kg (25th centile), with apparently normal length. Mild neonatal hypotonia was noted. Slender, generally hypermobile fingers were present, with bilateral contractures of the fifth digits preventing full extension. Skin hyperelasticity was reported in infancy. In keeping with her hypotonia, motor milestones were achieved slightly later than average. Language and social milestones were achieved at appropriate ages. No fractures, joint dislocations or developmental hip dysplasia were present. Hearing assessments showed no abnormality, but her mother noted she had a tendency to speak in a loud voice.

An echocardiogram at aged 3 performed in the context of difficult breathing and snoring showed mitral valve prolapse and mild mitral regurgitation with a normal aortic root diameter. She was prescribed enalapril until age 4 years which was then ceased after a follow up cardiac review.

At 4 years of age the child presented with spontaneous vertical Descemet's membrane splitting in the cornea of the right eye, resulting in corneal edema. The left cornea was noted to be thin with keratoconus and blue sclerae were noted. She subsequently sustained a corneal rupture in her left eye aged 4 years 3 months with subsequent total visual loss. She has very little residual vision in her right eye (3/60 m for distance and N18 for near vision according to Roman test type notation).

Pure tone audiometry at 4.5 years showed bilateral mild hearing loss and absent otoacoustic emissions above 2 kHz in both ears, suggesting abnormal cochlear outer hair cell function bilaterally in the high frequency tones. Masked bone conduction studies could not be performed but conductive hearing loss was further suspected in at least one ear, with associated high tone hearing loss (45 dB at 4000 Hz) without obvious middle ear pathology. Tympanometry was consistent with hypercompliant tympanic membranes, with hypermobility of the ossicular chain which suggested as a possible explanation for the conductive component.

Aged 4 years, the patient's height and weight were above average for her age (75th and 95th centiles, respectively) with no evident dysmorphism. Soft but not hyperextensible skin was noted, with no abnormal bruising or scarring. Generalized joint hypermobility, particularly in the fingers, was present, with a Beighton score of 8/9. She had prominent fetal fingertip pads and pes planus, but no pectus deformity or scoliosis.

Currently management includes the use of prophylactic eye goggles which are worn 24 h per day and lubricating topical eye drops to prevent eye rubbing of the eyes and reduce discomfort. She has bilateral hearing aids and good language skills. Corneal transplantation of both eyes has been performed and resulted in visual recovery in both eyes. Monitoring is ongoing.

Molecular investigation revealed homozygosity for a newly identified c.3476delG (p.Gly1159Alafs*105) mutation in *ZNF469*.

### 3.3 Patient 2 (P2)

This British Caucasian girl was 7 years old when last reviewed. She was blind in her left eye following repeated corneal ruptures, a failed donor graft operation, and secondary glaucoma which has required repeated applications of cyclodiode laser. She also had poor visual acuity in her right eye (3/24 for distance, and N8 for near vision according to Roman test type notation) due to extreme myopia (− 29 diopters) and early keratoconus.

She and her non-identical twin sister were the product of an in vitro fertilization pregnancy of British parents, delivered at 32 weeks gestation. She weighed 1.275 kg (3nd–10th centiles), and her sister weighed 1.588 kg (25th–50th centiles). Joint hypermobility was noted in early life, and a unilateral unstable hip was successfully treated conservatively using a harness for 6 months.

Aged 2 years, she was prescribed spectacles for significant myopia and was found to have blue sclerae upon referral to ophthalmology. A minor accident resulted in a penetrating corneal injury that was unsuccessfully repaired as the surgical sutures tore and subsequently detached from the corneal tissue. A donor corneal graft was subsequently applied but later became dislodged and severely damaged due to normal play activities. At the time of operation the left lens was noted to be lost, and a cataract present in the unperforated right eye. The left eye subsequently developed secondary glaucoma, treated with laser therapy, and has no residual vision. Progression of disease became apparent in the uninjured right eye, with progressive enlargement of the globe, increasing corneal ectasia and myopia. The patient permanently wears protective eyewear and was considered unsuitable for a scleral contact lens. Dorsolamide/timolol (Cosopt) drops were used in both eyes, and latanoprost (Xalatan) drops in the glaucomatous left eye.

Hearing loss had been diagnosed in early childhood. Repeated ear tube insertions and adenoidectomy gave some benefit, and she used bilateral hearing aids. Her developmental milestones were appropriate, given her combined sensory loss, and she was receiving additional help in mainstream school. No other family members had suffered corneal rupture, though her twin sister and younger brother (*ZNF469* genotypes not tested) both wore spectacles for strabismus and astigmatism respectively and had blue sclerae and mild joint hypermobility. Their parents had normal vision, and there was no other relevant family history.

The patient is compound heterozygous for two mutations in *ZNF469* c.[5788delC];[5788dupC] (p.[Gln1930Argfs*6];[Gln1930Profs*133]). These mutations were initially regarded as artefacts, however further investigations confirmed the finding in the patient, and revealed that the mother was a heterozygous carrier for c.[5788delC], while the father was a heterozygous carrier for the c.[5788dupC], arguing for a mutational hot spot.

### 3.4 Patient 3 (P3)

The 18 year-old female patient, whose technical details of corneal repair were previously reported by Hussin et al. [29], had visual acuity reduced to perception of hand movements bilaterally from the age of 11 years, following multiple bilateral corneal ruptures secondary to minimal trauma. She was the child of first cousin parents of Pakistani origin, and had two brothers with normal vision and no relevant family history. Hearing tests had never shown any abnormality. Soft, smooth skin and moderate joint laxity, with a Beighton score of 4/9, was demonstrable, with hypermobility that was most marked in the distal interphalangeal joints. Contractures of the 5th medial proximal interphalangeal joints resulted in progressive camptodactyly from the age of 3 years. Similarly, she had over-riding toes and required surgery for bilateral hallux valgus at aged 14 years. Slow wound healing had been noted. She suffered a skull fracture after trauma at the age of 7 months but has since sustained no other fractures. Her speech and motor development were delayed, with first walking at 30 months of age, but good progress thereafter.

On examination, markedly blue sclerae were present, as were extensive corneal scarring, keratoconus and keratoglobus. Aged 18, she was affected with secondary glaucoma and was totally blind; placement of bilateral prosthetic globes is therefore under consideration.

Molecular investigations revealed homozygosity for a previously unreported *ZNF469* mutation c.6444delG (p.Gln2149Serfs*51).

### 3.5 Biochemical analyses

Total urinary pyridinoline ratios (LP/HP) were within the normal range (mean 0.22, range 0.16–0.3, SD 0.04) when compared to those of the control samples (LP/HP 0.19 ± 0.02; 0.12–0.25; n = 179) [27].

### 3.6 Molecular analysis

**Fig. 1:**
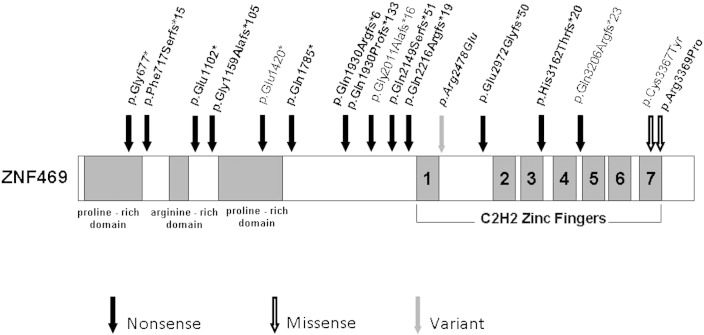
Schematic of ZNF469 protein, showing the proline-rich and arginine-rich domains, and the 7 C2H2 zinc fingers towards the C-terminus. The newly identified nonsense and missense mutations, and the SNP variant are depicted in bold. The 4 previously reported mutations are depicted with the updated nomenclature according to the finding showing that *ZNF469* is a single exon gene. Nonsense mutations are likely to evade nonsense mediated decay and predict a truncated protein.

A total of 23 patients from 13 unrelated families had molecular testing for *ZNF469* and subsequently *PRDM5*. Mutations in *ZNF469* and *PRDM5* were found in all patients but one (Table 1, Table 2, Fig. 1); 18 patients were either homozygous or compound heterozygous (P2) for *ZNF469* mutations (Table 1, Table 2) while, 4 were homozygous for *PRDM5* mutations as previously described [7]. All parents were tested for mutations and found to be carriers for the identified disease causing mutation. In particular, the mother of P2 is a heterozygous carrier for the c.5788delC mutation in *ZNF469*, while the father is a heterozygous carrier for the c.5788insC mutation *ZNF469*. The novel c.7508C>A (p.Arg2478Glu) SNP variant in *ZNF469* found in heterozygosity in 3 patients from two unrelated families (Fig. 1 and Table 2) is regarded as a normal variant based on computational analysis.

**Table 2:** Summary of the molecular findings on the entire BCS cohort described clinically by Al-Hussain et al. [1]. This includes mutations in both *ZNF469* and *PRDM5*. Six novel *ZNF469* homozygous mutations and one heterozygous SNP (reported in italics) are shown. Additionally, the two homozygous *ZNF469* mutations (^#^) as well as the three homozygous *PRDM5* mutations(**^§^**) reported previously [7] are given for completeness. Only patient 912_11 does not have a mutation in either *ZNF469* or *PRDM5*, suggesting further genetic heterogeneity in BCS.

Patient Nr.	*ZNF469*		*PRDM5*	
901_04			c.93 + 1G>A**^§^**	Aberrant splicing**^§^**
902_03	c.7508C>A	*p.Arg2478Glu*	c.320A>G**^§^**	p.Tyr107Cys**^§^**
903_03	c.6647delA	p.Gln2216Argfs*19		
903_04	c.6647delA	p.Gln2216Argfs*19		
904_03	c.8901_8914dup	p.Glu2972Glyfs*50		
904_04	c.8901_8914dup	p.Glu2972Glyfs*50		
904_08	c.8901_8914dup	p.Glu2972Glyfs*50		
908_04	c.3304G>T	p.Glu1102*		
909_06	c.5353C>T^#^	p.Gln1785*^#^		
909_07	c.5353C>T	p.Gln1785*		
910_03	c.2029G>T	p.Gly677*		
910_05	c.2029G>T	p.Gly677*		
912_11	*No mutation*		*No mutation*	
914_04	c.7508C>A	*p.Arg2478Glu*	c.1517–1527delTTCATATCCGG**^§^**	p.Val506Glufs*5**^§^**
914_06	c.7508C>A	*p.Arg2478Glu*	c.1517–1527delTTCATATCCGG**^§^**	p.Val506Glufs*5**^§^**
915_06	c.2150delT	p.Phe717Serfs*15		
915_07	c.2150delT^#^	p.Phe717Serfs*15^#^		
916_03	c.9483delG	p.His3162Thrfs*20		
917_09	c.5353C>T	p.Gln1785*		
918_06	c.10106G>C	p.Arg3369Pro

Our previous expression microarray analysis performed on dermal fibroblast cultures from two homozygous *PRDM5*-mutants (c.1768C>T and Δexons 9–14 [7]) and two *ZNF469* homozygous mutants (c.5353C>T patient 909_06 [1] and c.2150delT patient 915_07 [1]) respectively, demonstrated a significant transcriptional changes in genes involved in ECM regulation, in particular of *COL4A1*, *COL11A1*, *HAPLN1*, *EDIL3* and *TGFB2*, which were subsequently tested and validated by qPCR. This data suggested a molecular overlap between *ZNF469* and *PRDM5* mutant dermal fibroblasts [7]. Interestingly *PRDM5* transcripts were unaltered in either of the *ZNF469* mutant fibroblasts.

**Fig. 2:**
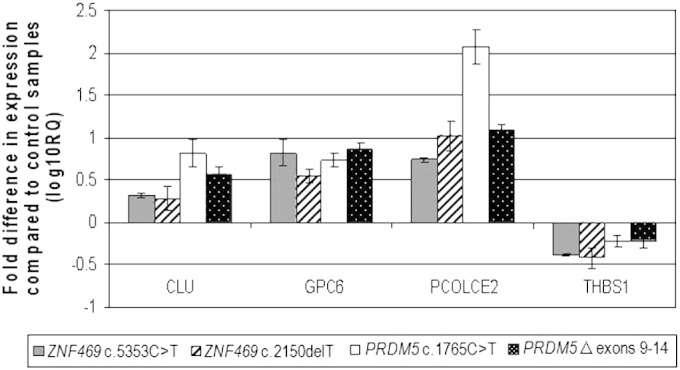
Quantitative RT-PCR assessment of target genes identified in microarray analysis. Fold changes in mRNA expression of genes highlighted in the microarray were assessed in dermal fibroblasts isolated from four BCS patients with different mutations: *ZNF469* c.5353C>T, *ZNF469* c.2150delT, *PRDM5* c.1765C>T, and *PRDM5* Δ exons 9–14. mRNA levels were normalized to *GAPDH* expression, and the fold change is displayed relative to 4 individually age, sex and ethnicity matched control fibroblast lines. The y axis represents fold changes (log10) in gene expression and the x axis the target assessed. Transcripts assessed were Clusterin (*CLU*), Glypican-6 (*GPC6*), Procollagen C-endopeptidase enhancer 2 (*PCOLCE2*) and Thrombospondin (*THBS1*). Error bars represent the standard error of the mean (n = 18 independent experiments). For each of the four genes assessed, transcript levels were significantly reduced and assessed by one-way ANOVA and Bartlett's post-test comparison (p < 0.0001).

In our present study four genes that previous expression microarray studies showed to be significantly altered in the two *ZNF469* mutants, compared to age and sex-matched controls (data not shown), were further investigated by qPCR in both *ZNF469* and *PRDM5* mutants, respectively (Fig. 2).

(i) Expression of *GPC6* was increased in the *ZNF469* mutant dermal cells compared to controls cells (< 2-fold). qPCR further corroborated the increased *GPC6* message levels in both *ZNF469* mutant cells as well as the two *PRDM5* mutant dermal fibroblast cultures (< 0.554-fold).
(ii) Elevated levels of *CLU* mRNA was detected in the *ZNF469* mutant dermal fibroblasts cultures (> 8-fold increase). Increase in *CLU* message in all 4 mutant dermal fibroblast cultures was observed by qPCR (> 0.282_log10_).
(iii) A 10-fold reduction in *THBS1* expression was observed in the c.2150delT *ZNF469* dermal fibroblasts microarray and qPCR validation confirmed the reduction of *THBS1* in both *ZNF469* mutant dermal cultures (c.2150delT *ZNF469* − 0.378_log10_ and c.5353C>T *ZNF469* − 0.415_log10_). Similarly, the *PRDM5* mutant fibroblast cells displayed a reduction in *THBS1* expression when assessed by qPCR (Δ 9–14 − 0.211_log10_ and c.1765C>T − 0.218_log10_).
(iv) *PCOLCE2* expression was increased by > 6-fold in both *ZNF469* dermal cultures when analyzed by microarray. Validation by qPCR observed that *PCOLCE2* expression was elevated in all 4 dermal lines (> 1.022_log10_).

In summary, the overlapping transcriptional profiles of fibroblasts with *PRDM5* or *ZNF469* mutations suggest a shared biological pathway (Fig. 2).

**Fig. 3:**
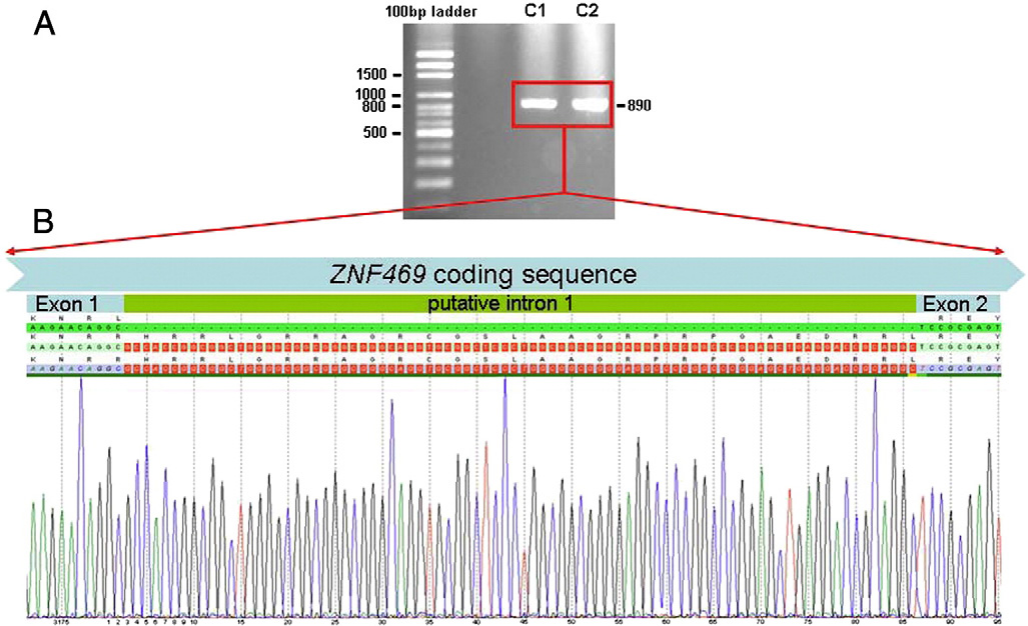
Evidence for *ZNF469* being a single exon gene. A. One-step RT-PCR of total RNA from two control fibroblast cultures amplifies an 890 bp fragment of *ZNF469* upstream and, respectively downstream the intron/exon boundaries. B. Sanger sequencing of both amplicons (only one is shown) shows a clean sequence which includes the previously annotated inton 1 sequence. This proves that the 84 bp intron is not spliced out.

Historically the *ZNF469* gene has been annotated and referred to as having two exons (Ensembl transcript ENST00000437464) [11], [30]. To ascertain whether the very short intron did exist, a primer pair upstream, respectively downstream the intron/exon boundaries was used for one-step RT-PCR of total RNA from two control fibroblast cultures. An amplicon of the size of 890 bp, that corresponded to *ZNF469* being a single exon gene, was amplified. Sanger sequencing confirmed that the putative 84 bp intron was not spliced out, confirming that *ZNF469* is a single exon gene (Fig. 3).

## 4 Discussion

Historically ZNF469 was considered as a poorly conserved zinc finger protein of unknown function. However, recently its influence on central corneal thickness has been repeatedly highlighted in genome wide association studies [23], [24], [26], [31], [32] and its role in ocular disease is of increasing interest. In this paper we report 10 novel mutations in *ZNF469* that cause BCS, further highlighting the role of ZNF469 in corneal development and integrity. We also show that the *ZNF469* amplicons amplified by RT-PCR included the intron sequence, suggesting that *ZNF469* is a single exon gene. Within our patient cohort, there is only a single patient who does not have a mutation in either *ZNF469* or *PRDM5* (912_11), despite typical clinical findings [1]. This suggests that the majority of cases of BCS are attributable to mutations in *ZNF469* or *PRDM5*, whilst it also suggests that there might be further genetic heterogeneity in BCS.

Molecular defects in ECM genes and their regulators tend to result in a failure of structural integrity and stability within affected organs and tissues [33], [34] as a consequence of disorganized synthesis, secretion or organization of ECM molecules. Most connective tissue disorders display some phenotypic overlap. Joint hypermobility, skin hyperelasticity and kyphoscoliosis are common overlapping features that have also been observed in a number of BCS patients [1], [7], [11].

The striking feature of BCS that differentiates it from a number of other connective tissue disorders is the severe, often blinding, ocular phenotype that may be present in the absence of extra-ocular features [35]. Compared to other connective tissue disorders that also display an ocular phenotype, such as EDS, osteogenesis imperfecta and Marfan syndrome, the systemic features of BCS are mild and not life-threatening. In particular, BCS presents clinical features overlapping with these of the kyphoscoliotic type of EDS (EDS VIA; MIM 225400) which is caused by the deficiency of the enzyme lysyl hydroxylase 1 (LH1). However, the following features are characteristic of EDS VI only, and may allow the two disorders to be differentiated on clinical grounds: severe muscular hypotonia at birth, progressive kyphoscoliosis starting in early infancy, fragility of the skin with abnormal scarring, a Marfanoid habitus, osteopenia, and rupture of the arteries. Since EDS VIA is biochemically diagnosed by a significantly increased ratio of lysyl pyridinoline (LP)/hydroxylysyl pyridinoline (HP) excreted in the urine [36], urinary pyridinoline analysis was performed in the BCS cohort to exclude EDS VIA.

In this context, it is important to remember that BCS patients are most likely to present to an ophthalmologist with high myopia, blue sclera, severe progressive corneal ectasia and/or a blinding ocular injury. In the absence of a family history or a previous corneal perforation the diagnosis can be easily overlooked and opportunities for anticipatory management in the form of reinforced polycarbonate spectacles missed. In addition, a number of families with BCS known to us have been investigated for non-accidental injury, in some cases leading to the removal of children from their parents. This further highlights the importance of raising awareness of this condition.

Patients with mutations in *ZNF469* or *PRDM5* display a near identical phenotype, suggesting that both proteins share the same biological pathway. We previously investigated gene expression in two fibroblast cell cultures with an internally deleted or truncated PRDM5 by microarray analysis [7] and went on to confirm changes by qPCR. The interesting targets were also assessed in two *ZNF469* mutant dermal cell cultures. All four mutants displayed very similar results with several key extracellular matrix components such as *COL11A1* and *EDIL3* being highly disrupted when assessed by qPCR [7]. The organization of the ECM was altered in all 4 BCS dermal cultures, further strengthening the hypothesis that ZNF469 and PRDM5 operate in the same biological pathway.

To complement and expand this work and to further elucidate the phenotypic similarities in BCS patients with *ZNF469* and *PRDM5* mutations, we compared the expression profile of ZNF469 mutant fibroblasts with those of *PRDM5* mutant fibroblasts for ECM-associated components of interest. Significant and comparable down-regulation of the ECM-associated genes was observed in the *PRDM5* and *ZNF469* mutants (Fig. 2) that would account for the connective tissue phenotype observed in BCS individuals. It is unknown how ZNF469 and PRDM5 regulate these key ECM genes, either through direct or indirect interactions.

The *ZNF469* gene was previously predicted to be a large two exon gene encoding a 13,203 bp transcript with a small in-frame intron. The putative intron is conserved in chimpanzee and gorilla, but poorly in other species. Splice site prediction programs do not identify the donor splice site and it is probable that the historical reporting of *ZNF469* as a two exon gene was due to incorrect annotation. Here, we have demonstrated that *ZNF469* is actually a single exon gene giving rise to a transcript of 13,279 bp (ENST00000565624). This finding means that mutations that were previously predicted to result in nonsense-mediated decay [11] are, in fact, likely to evade this pathway and result in the generation of a truncated protein (Fig. 1).

Further elucidation of the *ZNF469* and *PRDM5* pathway will provide significant insights into corneal development and maintenance, helping us to understand how their dysregulation leads to marked corneal thinning and dramatic fragility in BCS.
